# Principlism and contemporary ethical considerations for providers of transgender health care

**DOI:** 10.1080/26895269.2024.2303462

**Published:** 2024-01-19

**Authors:** Luke R. Allen, Noah Adams, Florence Ashley, Cody Dodd, Diane Ehrensaft, Lin Fraser, Maurice Garcia, Simona Giordano, Jamison Green, Thomas Johnson, Justin Penny, Katherine Rachlin, Jaimie Veale

**Affiliations:** a World Professional Association for Transgender Health Ethics Committee; b Independent Practice, Las Vegas, Nevada, USA; c Ontario Institute for Studies in Education, University of Toronto, Toronto, Ontario, Canada; d Center for Applied Transgender Studies, Chicago, Illinois, USA; e Transgender Professional Association for Transgender Health; f Faculty of Law, University of Alberta, Edmonton, Alberta, Canada; g Department of Psychiatry and Behavioral Sciences, University of Texas, Medical Branch at Galveston, Galveston, Texas, USA; h Department of Pediatrics, University of California San Francisco, San Francisco, California, USA; i Department of Urology, Cedars-Sinai Medical Center, Los Angeles, California, USA; j Centre for Social Ethics and Policy (CSEP), School of Social Sciences, The University of Manchester, Manchester, UK; k Independent Legal Scholar, Vancouver, WA, USA; l Department of Anthropology (Emeritus), California State University, Chico, California, USA; m Department of Family Medicine and Community Health, University of Minnesota, Minneapolis, Minnesota, USA; n Center for Bioethics, University of Minnesota, Minneapolis, Minnesota, USA; o School of Psychology, University of Waikato, Hamilton/Kirikiriroa, New Zealand/Aotearoa

**Keywords:** Autonomy, beneficence, ethics, justice, nonmaleficence, standards of care, transgender

## Abstract

***Background****:* Transgender health care is a subject of much debate among clinicians, political commentators, and policy-makers. While the World Professional Association for Transgender Health (WPATH) *Standards of Care* (*SOC*) establish clinical standards, these standards contain implied ethics but lack explicit focused discussion of ethical considerations in providing care. An ethics chapter in the *SOC* would enhance clinical guidelines.

***Aims****:* We aim to provide a valuable guide for healthcare professionals, and anyone interested in the ethical aspects of clinical support for gender diverse and transgender people of all ages. Recognizing that the WPATH is a global association, we address broad challenges. We offer a reflection on general ethical principles, providing conceptual tools for healthcare providers, patients, and families to navigate the specific challenges they might encounter in transgender health care, in line with WPATH’s worldwide mission and scope.

***Method****:* This article employs a descriptive analysis, and our framework of reference is the four principles of biomedical ethics: *respect for autonomy*, *beneficence*, *nonmaleficence*, and *justice*.

***Results****:* The article presents a discussion on the four ethical principles as applied to transgender health care. We address issues such as respect for patient autonomy in decision-making, the role of beneficence and nonmaleficence in clinical interventions, and the importance of justice in equitable treatment and access to care. Some of the ethical concerns we address in this article pertain to the current sociopolitical climate, where there has been increasing legal interference, internationally, for transgender and nonbinary people, particularly youth, seeking medical care.

***Discussion****:* We highlight the interplay between ethical principles and clinical practice, underscoring the need for ethical guidance in addressing the diverse challenges faced by healthcare providers and patients in transgender health care. We advocate for continuous refinement of ethical thinking to ensure that transgender health care is not only medically effective but also ethically sound.

The World Professional Association for Transgender Health (WPATH) *Standards of Care* (*SOC*) lack explicit discussion on ethical considerations that may arise in providing care to transgender people. However, a growing body of literature illustrates the complex ethical dilemmas that may arise in transgender health care (Adams et al., 2017; Ashley, 2022c; Bauer et al., 2019; Gerritse et al., 2021; Hann et al., 2017; Vincent, 2018). These dilemmas span a broad spectrum, encompassing concerns from the allocation of limited resources, the ethics related to influencing the regular onset and progression of puberty, the justification for surgical procedures on ostensibly healthy tissue, the appropriate authority to grant consent for treatments (be it the patient or the guardians), to the determination and interpretation of an individual’s best interests. In addition to these, the current sociopolitical climate shows instances of legal interference, internationally, for transgender and nonbinary people, particularly youth, seeking medical care (Arnold & McNamara, 2023). We analyze these questions using a framework known as principlism or the four principles approach, as this framework is among the most well-known in biomedical ethics (Shea, 2020a).

The WPATH *SOC* provides clinical guidelines: The *SOC* are not strictly prescriptive but allows for adaptability based upon the individual needs of the patient according to the legal norms of the jurisdiction wherein a patient may be receiving care. The *SOC* was the result of years of consultations, literature reviews, and clinical dialogue amongst expert clinicians from all areas of the world, and, as such, has retained over the decades the reputation as the most authoritative *SOC* in transgender health. However, the *SOC* (similar to other authoritative guidelines, such as the US Endocrine Society Clinical Guidance; Hembree et al., 2017) lacks an ethics chapter. WPATH is a worldwide association. Its scope transcends the boundaries of individual nations, and its mission speaks to all humans. To reflect the broad and universal scope of the mission of a worldwide association, in this article, we offer a broad reflection on general ethical principles which may provide useful conceptual tools to reflect on the specific challenges that providers of transgender health care, patients and families might encounter. Our analysis predominately offers an applied descriptive account of the four principles approach in transgender health care (i.e. we are less concerned with making absolute normative claims about how things ought to be, should be, or ought to be done). In line with the spirit of the mission of the WPATH, we wish to raise awareness of the moral dimension involved in gender care. Some of the challenges that patients and healthcare providers will encounter are inherently clinical; many, however, are ethical in nature. We hope to help the readers distinguish between ethical and clinical issues and to give a framework that can function as a broad moral compass, to examine and navigate the complexities that many might face. The framework that we will use for this purpose is “the four principles approach.”

## Principlism in clinical ethics

Principlism was first introduced in 1978 by the *Belmont Report*, which sought to develop common principles for research ethics (Friesen et al., 2017). Under principlism, ethical questions are informed by balancing four nonhierarchical principles (Beauchamp & Childress, 2019): *respect for autonomy*, *beneficence*, *nonmaleficence*, and *justice* for patients. Each principle must be weighed based on the particular elements of the ethical question being posed. Principlism offers a flexible set of principles that can be integrated with higher-level ethical frameworks like utilitarianism, deontology, and virtue ethics. Principlism does not aim to supplant more comprehensive moral theories but instead sets up a common ground (or, as Huxtable [2013] argues, a “starting point”) from which clinicians and ethicists may discuss and answer ethical questions. The application of principlism to a particular ethical question does not necessarily outweigh other important considerations, such as the rights of health care providers. It is important to acknowledge that providers also have a right to autonomy (Pellegrino, 1994) and that this autonomy may sometimes result in burdens for patients. Except where relevant due to the principle of justice or other applicable principles, we do not endeavor to address issues of provider rights in this article.

While principlism has proven to be influential, it has also faced numerous critiques, particularly regarding its application to complex and diverse contexts. Critics have pointed out that its roots in Western philosophy may not fully capture the ethical intricacies encountered in culturally and individually diverse contexts (Holm, 1995). There are also concerns about its potential to oversimplify ethical dilemmas, potentially overlooking the nuanced and multifaceted nature of real-world scenarios (Shea, 2020b). However, principlism remains a widely used framework in clinical ethics and in clinical practice. Clinical practices that obviously violate any of those principles are, prima facie, ethically problematic. National and international regulations often draw on principlism such as the United States Common Rule (found in various places in the Code of Federal Regulations, such as 45 CFR Part 46) or Canada’s *Tri-Council Policy Statement: Ethical Conduct for Research Involving Humans* (Canadian Institutes of Health Research et al., 2022). Principlism is also a core component of education in medicine, mental health disciplines, and human subject research (e.g. Mijaljica, 2014; United Nations Educational, Scientific, and Cultural Organization, 2016; World Health Organization, 2009, 2015). Therefore, this framework offers a method to quickly identify and examine ethical issues that is likely to be familiar to healthcare professionals and can thus have practical use. In the following sections, we discuss each of the four principles in relation to the practice of transgender health.

## Respect for autonomy

The principle of *respect for autonomy* affirms that patients have a right to decide among clinical options based on their own values, beliefs, and preferences (Beauchamp & Childress, 2019). Bodily autonomy and gender self-determination are emblematic of this right. One of the most important corollaries of the principle of respect for autonomy is the duty of clinicians to provide patients with the information necessary for them to make informed decisions. For a medical procedure to be ethical, the patient must provide free and informed consent (or assent). Though exceptions may be made in cases where the patient is incapable of providing informed consent or assent due to unconsciousness, mental incapacity, or emergency situations where delay in treatment may lead to harm or death.

Administering any medical treatment without or against the stated wishes of the patient is, in normal circumstances, a violation not only of their bodily integrity but also of their autonomy. Gender-affirming care (GAC), in this sense, is rather different from other areas of care (such as oncology or mental health), in that it is uncommon to have patients who refuse medical treatments that clinicians deem appropriate. We, therefore, do not usually witness, in this area of care, the ethical issues involved in cases in which clinicians attempt to impose medical treatments on patients against their wishes, for their own good. The more common scenario is one in which the patient approaches clinical services with a request or an outcome in mind, and the clinician must decide whether the request can or should be fulfilled. While clinicians may be ethically or legally obligated to provide only treatments that are beneficial and medically necessary, patients do not have a moral or legal right to receive treatments deemed nonbeneficial. However, in GAC, it is only through understanding the individual, their unique needs, and circumstances, and thus it is only through respecting their autonomy, that clinicians might form a reasonably accurate view of whether available treatments are likely to be beneficial. Therefore, while the principle of respect for autonomy does not dictate that clinicians abide by any request that is autonomously made, it does require them to engage in honest and frank discussions with patients about treatment options. This includes helping patients understand their options and the potential outcomes, and ensuring patients are fully aware of the risks and benefits of proposed treatments, as well as the uncertainties in medical knowledge (Kimberly et al., 2018).

## Beneficence & nonmaleficence

Beneficence involves an obligation on part of the provider to act for the benefit of others (Beauchamp & Childress, 2019), whereas the principle of nonmaleficence reflects the maxim “above all, do no harm” (Beauchamp & Childress, 2019). An intervention respects the principle of beneficence if, overall, it improves the welfare of the patient or conveys some other benefit to them. An intervention respects the principle of nonmaleficence to the degree to which it limits harms, side effects, and other unintended costs to the patient. These two principles are often considered together, in that clinical interventions are expected to maximize possible benefits and minimize possible burdens to the patient.

### Beneficence

The principle of beneficence is the source of many ethical duties in transgender health. Regardless of the setting or clinical population, health care providers must notably provide services in an affirming, supportive, and nonjudgmental manner (Hann et al., 2017). Facilitating access to GAC, such as by writing a referral letter for hormones, can be an important part of beneficence because access to care is linked with improved mental health and psychosocial functioning.

### Nonmaleficence

According to nonmaleficence, health care providers should not cause needless harm. Further, risks and adverse consequences associated with clinical practices must be justified by countervailing considerations that reflect respect for autonomy, beneficence, or justice. Arguments in favor of withholding or delaying access to care for transgender adolescents often appeal to the principle of nonmaleficence by asserting that the risks of adverse effects or regret associated with puberty blockers, hormones, or surgical interventions constitute harm (see Priest, 2019; Thompson, 2019). However, health care providers should be careful not to overstate the implications of the principle of nonmaleficence. The mere presence of risk is not by itself enough to justify denying treatment (Beauchamp & Childress, 2019). Few interventions in any health condition pose no risk at all.

### Weighing benefits and potential for harm

As with all four principles, risks and burdens must be weighed against the benefits of interventions and any other relevant consideration pertinent to respect for autonomy, beneficence, or justice (Varkey, 2021). For instance, concerns over bone density loss associated with puberty blockers must be weighed against respect for gender self-determination, potential mental health benefits, the possibility of avoiding future surgical interventions, and the potential harms of not providing treatment. Of the available evidence we have, bone density loss may be mitigated by subsequent hormone administration, increasing levels of physical activity, and dietary calcium (Lee et al., 2020). Nonetheless, scientific and developmental uncertainty can make it difficult to ascertain whether a given practice respects nonmaleficence and beneficence. In cases where the relative benefits and harms have not been well-studied, future research may determine that clinicians who withheld or delayed care may have negatively impacted the patient’s welfare. On the other hand, upon further study, interventions that initially showed promise may eventually be shown to have minimal intended benefits and many unintended costs or harms. When there are doubts about the short and long-term risks of a proposed medical intervention, a prudent approach is to proceed cautiously, giving weight to the patient’s wishes (autonomy), choosing treatments with potential benefits (beneficence), and starting with more reversible options to minimize unforeseen harm (nonmaleficence). Such a step-by-step approach helps in navigating ethical decision-making amidst scientific uncertainty, ensuring that the principles of autonomy, beneficence, and nonmaleficence are all given due consideration. However, it should be noted that gender self-determination and bodily autonomy, by definition, typically favor offering GAC.

### Justice

The principle of justice is concerned with fairness and equity in the distribution of benefits and burdens in society (Beauchamp & Childress, 2019). Although definitions of fairness and equity are subjective and open to substantial debate, in essence, the principle of justice suggests that people should not be treated differently when their past and present circumstances are equivalent. The principle of justice has both substantive and procedural aspects (Cookson & Dolan, 2000). As a substantive principle, justice requires the fair distribution of benefits and burdens associated with health care practices. As a procedural principle, it requires the fair and impartial application of established rules and regulations, such as when allocating health care resources. Justice as a procedural principle also requires health care providers to adequately hear and consider the perspective of patients and other stakeholders when making a decision and, notably, when weighing the principles of respect for autonomy, beneficence, and nonmaleficence.

Taking into consideration the principle of justice, in concomitance with the three other principles, can help clinicians navigate potential dilemmas or questions arising in transgender healthcare. For example, the principle of justice reminds us that GAC should not be subject to higher requirements (e.g. in terms of evidence base) or barriers than other forms of health care. Justice can also be served in other ways: for example, by offering a sliding scale or reduced fee structure for individuals who are experiencing financial difficulties. Justice can also be served by writing free referral letters for GAC or legal documentation changes, a practice often motivated by the economic precarity and low access to health care experienced by trans communities (American Psychological Association [APA], 2015; Kimberly et al., 2018; Watson et al., 2019). Other avenues for seeking justice include advocating for GAC coverage with private healthcare insurance companies and educating other healthcare professionals so that they can provide sensitive, respectful, and competent gender-informed care. In publicly funded healthcare systems, it is likely that some, but not all, the interventions requested by an individual, are covered by the State. Certain highly innovative procedures might not be funded at all. What the principle of justice also reminds us, however, is that the criteria for coverage should be consistent with the same procedural and substantial rules applied in other areas of healthcare.

## Common ethical questions, dilemmas, and areas of contention

In this section, we address common ethical questions, dilemmas, and areas of contention that arise in transgender health care. Our discussion includes questions relating to diminished capacity, pediatric and adolescent gender affirmation, individualized care, harm reduction, and BMI requirements. In the following, we assume that any interactions with health care providers are done through shared decision-making with all relevant stakeholders (e.g. patient, parents, and substitute decision-makers when appropriate), follow the WPATH *SOC* (Coleman et al., 2022), and utilize assent and consent practices such as those put forward by the American Academy of Pediatrics Committee on Bioethics (Katz et al., 2016). By shared decision-making, we mean a process where the client is the main decision-maker, fully informed by the clinician(s), with the healthcare provider acting as a technical expert, supporting the client’s autonomy and understanding of treatment choices (Gerritse et al., 2021; see also Coleman et al., 2022). For minors, this is typically done in close collaboration with, and the permission of, the parents.

## Diminished capacity

Typically, patients will be presumed to have the capacity to consent to, or to refuse, medical treatment at the age of majority in any given country. Some people may lack the capacity to make medical decisions due to their age, maturity, mental health, or cognitive impairments (Owen et al., 2009; Romero & Reingold, 2013; Woodhouse, 2003). Capacity is defined differently in different countries, and the implications of a finding of incapacity are also likely to vary significantly. Decision-making capacity can vary across time and lifespan based on the patient’s level of understanding, maturity, cognitive ability, emotional state, and environment. The capacity to consent and autonomy lie on a continuum, with people possessing varying degrees of each throughout their lives (Beauchamp & Childress, 2019).

Clinicians must typically follow different procedures when offering care to patients who do not have the capacity to consent to treatment. However, beneficial treatment typically should not be denied to those who are not able to consent. In those cases, the questions become how to obtain assent (to the degree possible), who can make medical decisions on their behalf, and how their best interests can be understood and served.

The case of GAC presents interesting challenges in this regard. It is through frank and candid dialogue that clinicians can understand the nature of the patient’s predicaments, their needs, and desired outcomes, and assess with them what treatments are more likely to offer the benefit at the lowest risks. The elaboration of one’s gender usually requires certain levels of introspection. Decisions around medical treatments similarly usually require a degree of reflection around the implications of presentation in a certain gender in the workplace, at home, at school, and so on. A healthcare provider may mistakenly view a lack of capacity to consent to treatment as an ipso facto lack of capacity to benefit from GAC or even to form an accurate view of one’s gender identity. However, people might lack the capacity to consent, but they are unlikely to lack a gender, and their gender can be congruent or not with the sex designated at birth. A person might still experience distress over their secondary sex characteristics, notwithstanding their lack of capacity to consent to treatment. More importantly, as we have discussed elsewhere, people with a lack of capacity to consent to treatment still have a right to access GAC as well as other medically necessary healthcare with assisted decision-making from their healthcare providers, family members, or surrogate decision-makers. Here again, the four principles can offer valuable guidance in reflecting and making decisions that concern those who do not have full capacity to make medical decisions.

The principle of justice suggests that people should not be treated differently when their circumstances are similar. Insofar as medical treatment should not be denied to people who can benefit from it, even if they lack capacity to consent, gender diverse people who lack capacity should not automatically be excluded as suitable candidates for medical treatment. On grounds of justice, as in other areas of care, the questions will be what is in the patient’s best interests, and who can make medical decisions on their behalf. Arriving at an answer to these questions might not be easy in some cases, but, again on grounds of justice, it should not be assumed that they cannot be answered. On grounds of autonomy, it must be acknowledged that patients might be unable to consent to treatment, but still be autonomous in expressing their gender. On grounds of beneficence, treatment that is likely to benefit a patient should not be withdrawn. On grounds of nonmaleficence, preventable suffering should be prevented. This does not, of course, mean that others are entitled to make decisions on behalf of a person with diminished capacity, regardless of what the person expresses or wishes. It means rather that ethical principles should be applied equally to gender diverse people, as they are applied to all others.

Pursuant to the principle of respect for autonomy, clinicians should strive to maximize the involvement of patients in decision-making by supporting their understanding and reasoning, and duly weighing their perspective. Information should be provided in a way that is accessible and appropriate to the patient’s level of understanding (APA, 2017; New South Wales Ministry of Health, 2020). An adapted, thoroughly informed consent process allows patients with diminished capacity to develop an awareness of the risks and benefits that they can expect from their decision to access GAC, which supports their ability to share their perspective, see it afforded due weight, and provide assent to care (Shumer & Tishelman, 2015).

### The case of minors

Identifying impediments to patients’ capacity to make health care decisions is an important part of the informed consent process (Kimberly et al., 2018). This is especially true when the patient is a minor. This view is consistent with legal and ethical guidance on medical decision-making for minors. According to the United Nations (1989) *Convention on the Rights of the Child* and as recognized by the American Academy of Pediatrics (Katz et al., 2016), youth should play an evolving role in decision-making as they age and become more mature. In many jurisdictions, the evolving autonomy of minors is recognized through the mature minor or *Gillick* doctrine, which grants minors legal capacity to minors to treatment when they are able to demonstrate sufficient maturity and understanding (Clark & Virani, 2021). Ashley (2022b) suggests that minors should have significantly more authority in decisions around GAC, even when they lack the capacity to provide informed consent, arguing that they are nevertheless better positioned than third parties to make decisions that strike at the heart of their personal identity.

Some empirical evidence suggests that many adolescents have comparable decision-making capabilities as young adults. Weithorn and Campbell’s (1982) study found that 14-year-olds did not significantly differ from 18- and 21-year-olds in their objectively-measured capacity to reason about and understand information about treatments for epilepsy, diabetes, depression, and enuresis. More recent research has been conducted with GAC in mind. Specifically, Vrouenraets et al.’s (2021) study found that 89.2% to 93.2% of trans adolescents (mean age, 14.71) were able to consent to puberty blockers. Studies on cisgender youths have shown that adolescents may be particularly prone to rash decision-making (see Blakemore & Robbins, 2012, for review). However, these concerns are context-dependent and likely to be significantly reduced when the decision is made in a calm environment with the support of parents and clinicians (Katz et al., 2016), which is generally the case in GAC. The American Academy of Pediatrics Committee on Bioethics (1995) warns against concentrating decision-making power in the hands of parents and clinicians, as it “diminishes the moral status” of minors (p. 316). When involving a substitute decision-maker, clinicians should ensure that they are capable and willing to act in the best interests of the child and ensure that the substitute decision-maker is not influenced by biases or moral objections that diverge from the patient’s best interests (Grimstad & Boskey, 2020).

Data shows a high prevalence of disorders on the autism spectrum among gender diverse young people, and concerns have been expressed about the ability of these young people to consent to treatment (Lim et al., 2022); it has also been argued that this cohort may be more confused about their self, and particularly prone to “social contagion” (Adams & Liang, 2020). On these grounds, some have recommended that young people with a diagnosis of autism wait longer or even indefinitely to receive GAC. It must be acknowledged that it is sensible for clinicians to be particularly cautious in offering medical treatment, when confronted with relatively unfamiliar situations, especially in the context of politically fraught interventions (e.g. Dewey & Gesbeck, 2017; Shuster, 2016). However, some of the concerns are not supported by current evidence, and clinicians should actively consult the empirical literature with regard to their concerns. For example, counter to concerns about “social contagion,” autistic patients may be less likely to be influenced by social pressures than nonautistic patients (Walsh et al., 2018; Wattel et al., 2022).

Again, the four principles here can assist in navigating some of the ethical decisions when working with minors and their parents. These principles remind us that the patient must be at the center of clinical concerns; therefore, however politically fraught, clinical practice should proceed with a view of serving the interests of patients, on grounds of beneficence. The principle of justice reminds us that people with gender dysphoria, even if they are young and experience co-occurring health issues, should not be treated differently from all others. Cognitive, physical, or intellectual disabilities do not, in and of themselves, automatically prevent capacity to consent or assent to treatment, and young people with a diagnosis of autism should not be assumed to lack capacity (Academic Autistic Spectrum Partnership in Research and Education, 2015; Beauchamp & Childress, 2019). Of course, some gender diverse young people might lack the ability to consent to treatment, and some people might not reach the threshold of legal competence. However, that does not entail that they cannot have a gender, and therefore gender incongruence or dysphoria. Consequently, it cannot be assumed that they, as a whole, cannot benefit from medical treatment. Any such assumption would lead clinicians to violate the principle of beneficence, which is at the heart of medical professions. The question then becomes whether treatment is likely to benefit the patient. If treatment, on careful consideration, is likely to benefit the patient, then it would be a violation of justice, beneficence and nonmaleficence to deny or delay it.

## Pediatric gender care

GAC for minors is an issue of concern to the parents or guardians (hereafter referred to as parents) of these minors. Unfortunately, GAC for minors is presently highly contentious, with a great deal of misinformation on the subject, and accompanying parental hesitancy regarding initiating it (Gill-Peterson, 2018). GAC clinicians therefore play a pivotal role in providing parents with the support and evidence-based guidance needed to support the minors’ autonomous decisions to begin this care (Allen et al., 2021; Kimberly et al., 2021; Quinn et al., 2018). Except for a small collection of case studies and qualitative reports, detailed information on the exact frequency with which parents and children disagree on aspects of GAC is largely not available (e.g. Allen et al., 2021; Healy & Allen, 2019; Kimberly et al., 2021; Quinn et al., 2018). The parent’s first line of disagreement may simply result in refusing to bring their child to a provider of GAC. This tactic is largely invisible to practitioners and quite effective, save for those minors with independent resources and of the age to make medical decisions alone. Hesitant parents may also seek out a GAC clinician for an assessment or opinion but disagree with their child on when or if they should initiate aspects of care. This clinical crossroads might partly arise from a fundamental difference in understanding regarding a minor’s capacity to comprehend their own gender identity.

It is not uncommon for parents to deny access to GAC due to their belief that their child is too young to know their gender (Ehrensaft, 2016). Some parents may also believe that their child is gender nonconforming rather than transgender and therefore does not require GAC. Others may believe that their child’s gender dysphoria will resolve if they are not affirmed in their expressed gender and do not receive GAC. There is some early evidence that gender dysphoria diagnosed before puberty typically does not persist after the onset of puberty (Kaltiala-Heino et al., 2018). However, this research has been robustly criticized for including and failing to distinguish between individuals who were gender nonconfirming (e.g. in dress or behavior), those who expressed a different gender identity from that assigned at birth, and those who explicitly articulated a desire for GAC (Ashley, 2022b; Ehrensaft, 2016; Karrington, 2022; Priest, 2019; Temple Newhook et al., 2018). Additionally, these early studies focus, by definition, on the experience of prepubertal children, for whom GAC would consist of social affirmation only. They, therefore, have little direct impact on the ethics of providing GAC to adolescents who have largely consolidated their gender identity following the onset of puberty (Ashley, 2022b).

More recent research contradicts the claim that gender dysphoria in children typically resolves with time (e.g. de Castro et al., 2022; Olson et al., 2022). In one study (*n* = 317), 2.5% of prepubertal children who had socially transitioned re-identified with their gender assigned at birth by the 5-year mark and, of these, none spontaneously expressed regret over initial transitions (Durwood et al., 2022). Other research suggests that transgender minors, like their cisgender peers, are often aware of their gender identity from an early age (Hässler et al., 2022; Olson et al., 2015). Zaliznyak et al. (2020), for instance, found that approximately 75% of transgender adults that went on to seek surgical GAC reported experiencing signs of gender dysphoria for the first time between the ages of three and seven.

Many minors question their gender identity and roles throughout their childhood and adolescence. Some will go on to be cisgender, while others will continue to explore their gender and may choose to access GAC. Children’s gender journeys are often not linear, and care should be taken against assumptions regarding how their relationship to gender will evolve (Kuper et al., 2019). There is currently no evidence-based means to identify and separate minors who may come to regret GAC from those who will not (Ashley, 2022c). Regret appears to be very low or low (Coleman et al., 2022; Durwood et al., 2022), perhaps due to the strict criteria used in the delivery of medical care. Withholding gender-affirming care appears to pose greater mental health risks than offering it (Priest, 2019). This is especially true given the social and developmental importance of adolescence and the distress transgender minors may experience from an irreversible endogenous puberty that results in physical developments that can be psychologically and financially costly to reverse. In weighing the consequences of delayed or denied pediatric gender care against the backdrop of diverse parental and societal views, the need for ethical guidance becomes evident. There is also an overall lack of data or targeted ethical guidance on the provision of pediatric GAC in situations of disagreement between parents and minor clients (Kimberly et al., 2021). Despite this, the informal consensus from clinicians appears to lean toward declining to offer or prolonging the initiation of this care if or until these issues can be resolved (e.g. Quinn et al., 2018). Clinicians’ reasons for doing so vary. They may, for instance, simply prefer not to override the wishes of parents or guardians; or, where the clinician may be inclined toward supporting the child’s desire over their parents, legal resources to do so may be inaccessible.

### Social transition in children: justice and autonomy

Blanket restrictions on social transition deny youths experiences that may be critical in exploring and/or consolidating their gender identity and could reinforce social messaging that encourages being cisgender (Ashley, 2019). Children often have to surmount significant barriers to expressing a transgender identity due to the presence of significant social messaging against being transgender and gender transition (Kidd et al., 2021). In this context, respecting a child’s developing autonomy might mean recognizing and supporting their self-expressed identity without imposing external assumptions about a particular gender trajectory. Delaying social transition may privilege cisgender identities over transgender ones. Since these concerns apply symmetrically, the principle of justice prohibits any double standards. Moreover, justice affirms not only equality in treatment but also an active effort to support children in expressing or exploring their gender identity safely without fear or shame. Wedding the principles of justice and respect for autonomy, clinicians should encourage parents to follow their child’s lead regarding social transition (Ehrensaft, 2011). They can do so by helping parents create a safe and supportive environment for their children to explore and express their gender identity and choices regarding social transition regardless of age (Coleman et al., 2022, Chapter 7; Ehrensaft, 2011; St. Amand & Ehrensaft, 2018). This approach aligns with a balanced view of respecting both the child’s autonomy and their evolving capacity for decision-making. When the child’s desire for social transition becomes clear, children should be afforded the freedom to explore and live in the social gender of their choice in a safe and supportive environment regardless of their age (Coleman et al., 2022, Chapter 7; St. Amand & Ehrensaft, 2018).

### Puberty blockers: autonomy, beneficence, nonmaleficence, and justice

In recent decades, puberty blockers have become increasingly available to minors who are “persistent, insistent, and consistent” in their gender dysphoria (Chung et al., 2020). As with social transition, it has been suggested that puberty blockers may deny youths important socio-sexual experiences associated with endogenous puberty that would lead them to re-identify with their gender assigned at birth (Giovanardi, 2017; Korte et al., 2008). The possibility of regret due to diminished autonomy, or perhaps the privileging of nonmaleficence over autonomy, is also cited as a reason to restrict access to puberty blockers (Strand & Jones, 2021). The principles of justice and respect for autonomy apply here similarly to social transition and puberty blockers. That is, while endogenous puberty may inhibit gender exploration by encouraging the child toward a cisgender identity, it will also force others to go through an irreversible and potentially distressing endogenous puberty that is at odds with their gender identity. Failing to provide treatment is not in itself a neutral act.

Access to puberty blockers has also been opposed on the grounds that they delay the maturation of gonads, contributing to later infertility, and pubescent youths cannot validly consent to infertility. In fact, current scientific evidence indicates that puberty blockers only lead to permanent infertility if they are administered in early puberty and followed by a gonadectomy (Krishna et al., 2019). Without a gonadectomy, in the absence of hormone therapy, it is possible to undergo gonadal maturation at any time simply by ceasing puberty blockers as their body may resume the process of gonadal maturation based on their endogenous hormonal milieu. Regardless, potential fertility considerations do not alone justify withholding puberty blockers, though clinicians should offer fertility and family planning counseling to all youth who request puberty blockers and/or hormone therapy (Coleman et al., 2022, Chapter 16). Youthful age does not automatically prevent mature reasoning and insight regarding infertility and future family-building desires. When a patient experiences diminished capacity to consent or assent, fewer ethical principles are compromised by waiting and exploring the possibility of undergoing endogenous puberty for fertility purposes at an older age rather than forcing the child to undergo endogenous puberty at a younger age. Such an approach better respects the child’s autonomy. When discussing the impacts of gender-affirming care on fertility, clinicians should be careful not to import their own biases and assumptions about family-building desires (Clark, 2021).

The practice of only offering puberty blockers to youths who display a persistent, insistent, and consistent history of gender dysphoria is inconsistent with the underlying justification of puberty blockers, which is to give youths more time to explore their gender identity before undergoing partially irreversible pubertal development. Since puberty blockers are about making time, it can be argued that they should be available to all youths who are questioning their gender and/or desire for GAC (see Wenner & George, 2021). There is no clear inherent violation of the four principles of biomedical ethics in offering puberty blockers to youths who request them, are able to provide meaningful assent, and can either consent on their own or obtain the permission of a substitute legal decision-maker. Further restricting access to puberty blockers for transgender and gender-questioning youths may be contrary to the principle of justice since puberty blockers are widely available for central precocious puberty without an evaluation of gender identity (Fuqua, 2013).

### Ethical issues in the provision of gender-affirming hormones (GAH)

Ethical concerns about the provision of GAH to adolescents often stem from its irreversible changes. If the patient were to later change their mind, they might regret these changes. The role of clinicians is not to determine whether an adolescent should pursue gender-affirming care, but rather to facilitate a collaborative decision-making process involving the patient and their parents. The decision of whether and when to initiate hormone therapy is an intensely personal and value-laden one, and it should primarily rest with the patient, who is often assisted by their parents in these matters. Rather than deciding for the adolescent, clinicians should offer guidance, support, information, and a nonjudgmental space for the youth (and their parents) to explore worries and doubts, weigh the risks and benefits associated with GAC, and decide in a way that best reflects their desires, values, and priorities. The possibility of regretting treatment does not alone provide moral grounds for denying it. If the potential for regret dictated medical decisions, many other treatments that are later regretted or discontinued would also have to be withheld, which would compromise the principle of justice that supports equitable healthcare.

While some concerns regarding offering GAH to adolescents relate to the risks of regret or detransition, especially due to its partially irreversible nature, these risks are often overstated, sometimes due to sensationalistic media coverage (MacKinnon et al., 2021). While there appears to be a numerical rise in the number of people who detransition, there is no evidence that the percentage of people who detransition and/or regret has been rising. In fact, because GAC has become more accessible in the last decade, we would expect a corresponding rise in the number of people who detransition. Furthermore, regret and detransition are not necessarily interlinked, and indeed, most who detransition do not express regret (Durwood et al., 2022; Sansfaçon et al., 2023; Turban et al., 2021). In any case, recent research indicates that detransitioning and regret remain rare in GAC relative to other medical interventions (de Castro et al., 2022; Hannema et al., 2022). Indeed, GAC interventions are consistently associated with either mental health benefits or neutral outcomes (Achille et al., 2020; Allen et al., 2019; Chen et al., 2023; Chew et al., 2018; de Lara et al., 2020; Grannis et al., 2021; Green et al., 2022; Kaltiala et al., 2020; Kuper et al., 2020; Tordoff et al., 2022; Turban et al., 2022). On the other hand, negative outcomes to mental and physical health are significant for adolescents who go on to transition as adults despite being denied GAC as adolescents (Costa & Colizzi, 2016).

The principle of nonmaleficence reminds us that harm can come from both providing and delaying or withholding treatment. The current research on GAC, particularly regarding hormone therapy for adolescents, is limited due to the practical and ethical challenges associated with conducting randomized-controlled trials. However, the strict necessity of demonstrating benefits of such care is debatable as access to GAC is closely linked to the fundamental right of gender self-determination (i.e. respect for autonomy). For instance, practitioners widely believe that reproductive health care should be offered regardless of proven mental health benefits (Ashley, 2022a). From this perspective, and assuming that the adolescent can provide meaningful assent or consent, one could argue that GAH should not be denied unless there is clear evidence of more harm than benefit. This stance is grounded in the principle of respect for autonomy and is also reflective of the principle of justice. Denying adolescents this form of care deprives them of the chance to experience puberty in a way that aligns with their gender identity. It also prevents them from reaching various social milestones at the same time as their peers. The bioethical principles form a framework for navigating decision-making under conditions of epistemic uncertainty regarding the long-term effects of treatment. These principles prompt careful consideration of the adolescent’s emerging autonomy and the potential benefits and risks of treatment, ensuring that patient care decisions, typically made in conjunction with family participation, are aligned with what is currently understood to be in the best interest of the adolescent.

## Evaluations and letters of support for surgery

The WPATH *SOC* recommends that patients undergoing gender-affirming surgeries have an assessment (depending on the surgery) from a health care provider experienced in gender care (Coleman et al., 2022). These assessments confirm the presence of diagnostic criteria (in those regions where it is required) and that the patient understands the nature of, and the anticipated risks and benefits of the surgery that they seek to undergo.

A letter of support is typically required prior to the provision of transitional medical interventions and for insurance coverage (Coleman et al., 2022). The professionals responsible for drafting this letter may experience tension between respecting their client’s autonomy and upholding the principle of nonmaleficence (i.e. not causing harm), which the letter is intended to mitigate. There are also implications for the principle of justice as locating a clinician willing and sufficiently trained to provide an evaluation can be costly and burdensome (Brown et al., 2020; Snow et al., 2022), reducing access to care for those with limited resources. Gender-affirming practitioners who wish to reduce barriers for transgender and gender diverse patients and minimize the extent to which they act as a gatekeeper might therefore perform a perfunctory assessment. In fact, there is no evidence to suggest that assessment of gender is effective at preventing decisional or outcome regret (Ashley et al., 2023). However, both the principles of nonmaleficence and autonomy require that the client have all the necessary information required to make a fully informed decision (see Ross et al., 2023). In this way, an assessment can function to uphold nonmaleficence and autonomy. For example, patients may not be aware of all surgical options or know the rates of complications associated with genital confirmation surgeries. They may be unaware that exposure to sunlight increases scar pigmentation or of the ways in which identity may shift after hormonal or surgical intervention (e.g. Davis & St. Amand, 2014). Examples of key areas to assess include ensuring the patient has the means to undergo follow-up visits with their surgeon, that they have realistic, reasonable expectations about their surgery, and that they have made adequate after-care and recovery plans. So, while an assessment need not be overly stringent, it should not be perfunctory. The professional responsible for drafting the letter can use the opportunity to assist in ensuring informed consent.

## Individualized embodiment care

Trans communities and clinicians are increasingly conceptualizing GAC as something that should be adapted to each patient’s individualized desires and embodiment goals, even if they do not conform to traditional GAC practices or gender norms and expectations. Examples of newer and more individualized GAC include but are not limited to low-dose hormone therapy (Cocchetti et al., 2020), selective estrogen receptor modulators (Xu et al., 2021), simultaneous testosterone and estrogen, surgery without hormone therapy (Vincent, 2019), and estrogen with mastectomy to remove unwanted breast growth (Cocchetti et al., 2020). Examples within GAC surgery include removal of the nipple during mastectomy, penile preservation vaginoplasty (QueerDoc, 2022), zero-depth vaginoplasty (Stelmar et al., 2023), and metoidioplasty or phalloplasty with vaginal canal preservation (Chen et al., 2021). Despite a growing recognition that gender-affirming care should be individualized, many of these options remain difficult to access due to practitioner unfamiliarity, discomfort with uncertainty, or fears of causing harm. The research literature on less common forms of gender-affirming care is presently quite limited.

Non-individualized approaches to gender-affirming care pose challenges due to the conflict between the patient’s right to gender self-determination and bodily autonomy and clinicians’ reluctance to provide services individualized to the patient’s embodiment goals, whether due to prejudice, scientific uncertainty, lack of education, inexperience, or insufficient technical expertise. Clinicians may also face these challenges with patients who seek out gender-affirming care while identifying as cisgender, which is known to occur with eunuch-identified individuals pursuing genital surgeries or cisgender masculine lesbians who request mastectomies to alleviate gender dysphoria and retain a female identity (Coleman et al., 2022, Chapter 9).

Pursuant to the principles of respect for autonomy and justice, clinicians should provide individualized GAC to the extent of their knowledge about all existing *available* options. Clinicians should also pursue education, training, and research to expand the range of gender-affirming interventions that they are able to safely and reliably offer. Patients have a right to gender self-determination and bodily autonomy. Denying care *solely* because their desires do not conform to prevailing gender norms and expectations, absent evidence that the requested intervention poses severe risks of harm, would compromise the principle of respect for autonomy. Social and medical GAC may produce benefits not because they align with gendered norms and expectations but rather because they reflect the person’s sense of gender and embodiment goals, thereby contributing to self-actualization, generating gender euphoria, and alleviating gender dysphoria. It follows, then, that nontraditional, newer, and innovative forms of gender-affirming care may produce similar benefits (Coleman et al., 2022, Chapter 8).

## Withholding care, harm reduction, and regret

Withholding GAC poses ethical problems from a harm reduction perspective. Someone who is denied GAC by a qualified health care professional may turn to unlicensed sources of hormone therapy or surgery out of desperation, subjecting themselves to unnecessary risks (Coleman et al., 2022; Mepham et al., 2014). Competent providers who are not specialized in GAC often decline to prescribe hormone therapy because they feel insufficiently knowledgeable or qualified (McPhail et al., 2016), which can lead to nonprescribed and unmonitored use of hormone therapy, especially in rural areas where clinicians specialized in GAC are not readily available. Risks associated with unlicensed care are heightened among people who cannot convince clinicians that they are “truly” trans or who desire atypical forms of GAC. Some eunuch-identified people have been reported to cause extremely painful and dangerous damage to their testicles in order to secure surgery from a licensed surgeon (Johnson & Irwig, 2014).

Under the principles of beneficence and nonmaleficence, clinicians should avoid barriers to care that may lead someone to secure GAC interventions from unlicensed sources. If health care providers are “competent to deliver similar care for cisgender patients, they should develop competency in caring for [transgender] patients” (Coleman et al., 2022, p. S143). While fears that a patient may regret a procedure are understandable, they do not outweigh the patient’s welfare or autonomy. GAC interventions pose unique health risks from unlicensed care because of their highly desired nature. Clinicians must appropriately consider these risks when offering or denying gender-affirming care.

Harm reduction perspectives on GAC are particularly important in light of the relative rarity of regret and the impossibility of predicting how a person will feel about interventions long into the future (Human Rights Campaign et al., 2016; McQueen, 2017; Michel et al., 2002; Rosenthal, 2014). Multiple bioethicists have suggested that current levels of regret are an unavoidable corollary of the unique and transformational nature of GAC rather than evidence of failure in clinical decision-making (Howard, 2022; McQueen, 2017). According to this view, the risk of harms from being withheld care are unnecessary and barriers to care would, therefore, violate the principle of nonmaleficence in addition to the principles of respect for autonomy and justice.

## Body mass index (BMI) requirements

Weight restrictions for GAC surgeries, such as BMI requirements, remain controversial and require careful consideration of ethical principles (Castle et al., 2023). One recent article reported that a quarter of transgender individuals presenting for GAC surgery are denied due to obesity (Taormina et al., 2023). On the one hand, these BMI-based restrictions to GAC may protect patients from an increased risk of complications and adverse health outcomes associated with surgery and postoperative care (Ives et al., 2019). For instance, some patients with very high BMI may be physically unable to dilate due to limited reach. On the other hand, weight requirements may unfairly disadvantage some patients. They can act as an insurmountable barrier to care, especially if they are used, as is common, in candidacy assessments for GAC (Castle et al., 2023; Taormina et al., 2023). Indeed, the idea that there is a predictive relationship between a BMI <30 and better GAC surgical outcomes is, at best, uncertain (Brownstone et al., 2021; Castle et al., 2023). Clinicians reconsidering weight restrictions should balance the risks of surgery for someone who has a higher BMI against the potential harms, impact on autonomy, and implications for justice of not proceeding with the surgery (Castle et al., 2022). The principle of respect for autonomy can justify a surgeon’s decision to offer surgery to patients with higher BMIs, so long as the patient is adequately informed of the relative and comparative risks of having surgery, pursuing weight loss, and foregoing surgery (Castle et al., 2022). Clinicians should have open discussions of weight-related risks with the patient and engage in shared decision-making with them, acknowledging that different surgeons and patients will have differing levels of tolerance for risk (Castle et al., 2022). If the patient elects to pursue weight loss, the clinician should adopt a patient-centered approach that does not reinforce weight stigma, focuses on healthy diet and lifestyle changes, and minimizes invasiveness in the patient’s life (Castle et al., 2022; Brownstone et al., 2021). Clinicians must remain conscious of the negative impacts of weight stigma on patients, and keep in mind that BMI requirements may result in tension with the principle of justice due to disproportionately impacting disabled communities and Black, Indigenous, and other people of color (Carender et al., 2022; Castle et al., 2023). In fact, there is evidence that BMI cutoffs for obesity differ depending on race/ethnicity (Rao et al., 2015).

## Conclusion

Guided by the principlism framework, we have explored common ethical dilemmas that present themselves in transgender health care. Despite the absence of a specific ethics chapter in the WPATH *SOC*8, we have illustrated the vital role of ethical principles in addressing issues related to equitable access to health, informed consent, and interpreting best interests within the complex tapestry of legal challenges and cultural diversity. The principles of respect for autonomy, beneficence, nonmaleficence, and justice serve as lenses through which these dilemmas can be viewed and navigated. Our discussion has underscored the nuanced ethical considerations in pediatric care, surgery evaluations, individualized embodiment care, and BMI requirements in GAC. These scenarios reveal the intricate interplay between respecting patient autonomy, ensuring beneficence, avoiding harm, and upholding justice. They highlight the importance of informed consent, the need for evidence-based guidance, the value of individualized care, and the importance of addressing barriers to access.

However, we also acknowledge that principlism, while a foundational guide, is not without its limitations. Its roots in Western philosophy and potential oversimplification of complex scenarios necessitate a cautious and context-sensitive application. Transgender health care, with its diverse and dynamic challenges, requires an ethical approach that is both principled and flexible, capable of adapting to individual needs and cultural contexts. As the field moves forward, we encourage clinicians and other stakeholders to continuously refine their ethical thinking, taking into consideration the evolving landscape of transgender health care, the diverse voices of those it serves, and the ongoing advancements in medical knowledge and practice. By doing so, we can help ensure that transgender health care remains not only medically effective but also ethically sound, equitable, and respectful of the rich tapestry of human experiences and identities.

## Ethics approval

This article does not contain any studies with human participants or animals performed by any of the authors.

## References

[CIT0001] Academic Autistic Spectrum Partnership in Research and Education. (2015). *AASPIRE healthcare toolkit*. https://autismandhealth.org/inc/content/pv_con-con_con.pdf

[CIT0002] Achille, C., Taggart, T., Eaton, N. R., Osipoff, J., Tafuri, K., Lane, A., & Wilson, T. A. (2020). Longitudinal impact of gender-affirming endocrine intervention on the mental health and well-being of transgender youths: Preliminary results. *International Journal of Pediatric Endocrinology*, *2020*, 8. 10.1186/s13633-020-00078-2 32368216 PMC7191719

[CIT0003] Adams, N., & Liang, B. (2020). *Trans and autistic: Stories from life at the intersection*. Jessica Kingsley.

[CIT0004] Adams, N., Pearce, R., Veale, J., Radix, A., Castro, D., Sarkar, A., & Thom, K. C. (2017). Guidance and ethical considerations for undertaking transgender health research and institutional review boards adjudicating this research. *Transgender Health*, *2*(1), 165–175. 10.1089/trgh.2017.0012 29098202 PMC5665092

[CIT0005] Allen, L. R., Dodd, C. G., & Moser, C. N. (2021). Gender-affirming psychological assessment with youth and families: A mixed-methods examination. *Practice Innovations*, *6*(3), 159–170. 10.1037/pri0000148

[CIT0006] Allen, L. R., Watson, L. B., Egan, A. M., & Moser, C. N. (2019). Well-being and suicidality among transgender youth after gender-affirming hormones. *Clinical Practice in Pediatric Psychology*, *7*(3), 302–311. 10.1037/cpp0000288

[CIT0007] American Academy of Pediatrics Committee on Bioethics. (1995). Informed consent, parental permission, and assent in pediatric practice. *Pediatrics*, *95*(2), 314–317. 10.1542/peds.95.2.314 7838658

[CIT0008] American Psychological Association. (2015). Guidelines for psychological practice with transgender and gender nonconforming people. *The American Psychologist*, *70*(9), 832–864. 10.1037/a0039906 26653312

[CIT0009] American Psychological Association (2017). Ethical principles of psychologists and code of conduct. https://www.apa.org/ethics/code/

[CIT0010] Arnold, J. C., & McNamara, M. (2023). Transgender and gender-diverse youth: An update on standard medical treatments for gender dysphoria and the sociopolitical climate. *Current Opinion in Pediatrics*, *35*(4), 423–429. 10.1097/mop.0000000000001256 37097294

[CIT0011] Ashley, F. (2019). Gatekeeping hormone replacement therapy for transgender patients is dehumanising. *Journal of Medical Ethics*, *45*(7), 480–482. 10.1136/medethics-2018-105293 30988174

[CIT0012] Ashley, F. (2022a). Adolescent medical transition is ethical: An analogy with reproductive health. *Kennedy Institute of Ethics Journal*, *32*(2), 127–171. 10.1353/ken.2022.0010 35815503

[CIT0013] Ashley, F. (2022b). The clinical irrelevance of “desistance” research for transgender and gender creative youth. *Psychology of Sexual Orientation and Gender Diversity*, *9*(4), 387–397. 10.1037/sgd0000504

[CIT0014] Ashley, F. (2022c). Youth should decide: The principle of subsidiarity in paediatric transgender healthcare. *Journal of Medical Ethics*, *49*(2), 110–114. 10.1136/medethics-2021-107820 35131805

[CIT0015] Ashley, F., Parsa, N., Kus, t., & MacKinnon, K. R. (2023). Do gender assessments prevent regret in transgender healthcare? A narrative review. *Psychology of Sexual Orientation and Gender Diversity.* Advance online publication. 10.1037/sgd0000672

[CIT0016] Bauer, G., Devor, A., Heinz, m., Marshall, Z., Pullen Sansfaçon, A., & Pyne, J. (2019). *CPATH ethical guidelines for research involving transgender people & communities*. Canadian Professional Association for Transgender Health. https://cpath.ca/wp-content/uploads/2019/08/CPATH-Ethical-Guidelines-EN.pdf

[CIT0017] Beauchamp, T. L., & Childress, J. F. (2019). *Principles of biomedical ethics* (8th ed.). Oxford University Press.

[CIT0018] Blakemore, S. J., & Robbins, T. W. (2012). Decision-making in the adolescent brain. *Nature Neuroscience*, *15*(9), 1184–1191. 10.1038/nn.3177 22929913

[CIT0019] Brown, H. M., Rostosky, S. S., Reese, R. J., Gunderson, C. J., Kwok, C., & Ryser-Oatman, T. (2020). Blessing or BS? The therapy experiences of transgender and gender nonconforming clients obtaining referral letters for gender affirming medical treatment. *Professional Psychology: Research and Practice*, *51*(6), 571–579. 10.1037/pro0000274

[CIT0020] Brownstone, L. M., DeRieux, J., Kelly, D. A., Sumlin, L. J., & Gaudiani, J. L. (2021). Body mass index requirements for gender-affirming surgeries are not empirically based. *Transgender Health*, *6*(3), 121–124. 10.1089/trgh.2020.0068 34414267 PMC8363993

[CIT0021] Canadian Institutes of Health Research, Natural Sciences and Engineering Research Council of Canada, & Social Sciences and Humanities Research Council of Canada. (2022). *Tri-council policy statement: Ethical conduct for research involving humans*. https://ethics.gc.ca/eng/policy-politique_tcps2-eptc2_2018.html

[CIT0022] Carender, C. N., DeMik, D. E., Elkins, J. M., Brown, T. S., & Bedard, N. A. (2022). Are bodymass index cutoffs creating racial, ethnic, and gender disparities in eligibility for primary total hip and knee arthroplasty? *The Journal of Arthroplasty*, *37*(6), 1009–1016. 10.1016/j.arth.2022.02.013 35182664

[CIT0023] Castle, E., Blasdel, G., Shakir, N. A., Zhao, L. C., & Bluebond-Langner, R. (2022). Weight stigma mitigating approaches to gender-affirming genital surgery. *Plastic and Aesthetic Research*, *9*(3), 20. 10.20517/2347-9264.2021.130

[CIT0024] Castle, E., Kimberly, L., Blasdel, G., Parker, A., Bluebond-Langner, R., & Zhao, L. C. (2023). Should BMI help determine gender-affirming surgery candidacy? *AMA Journal of Ethics*, *25*(7), e496–e506. 10.1001/amajethics.2023.496 37432002

[CIT0025] Chen, D., Berona, J., Chan, Y. M., Ehrensaft, D., Garofalo, R., Hidalgo, M. A., Rosenthal, S. M., Tishelman, A. C., & Olson-Kennedy, J. (2023). Psychosocial functioning in transgender youth after 2 years of hormones. *The New England Journal of Medicine*, *388*(3), 240–250. 10.1056/NEJMoa2206297 36652355 PMC10081536

[CIT0026] Chen, W., Cylinder, I., Najafian, A., Dugi, D., & Berli, J. (2021). An option for shaft-only gender-affirming phalloplasty: Vaginal preservation and vulvoscrotoplasty. A technical description. *Plastic and Reconstructive Surgery*, *147*(2), 480–483. 10.1097/prs.0000000000007579 33565834

[CIT0027] Chew, D., Anderson, J., Williams, K., May, T., & Pang, K. (2018). Hormonal treatment in young people with gender dysphoria: A systematic review. *Pediatrics*, *141*(4), e20173742. 10.1542/peds.2017-3742 29514975

[CIT0028] Chung, K., Rhoads, S., Rolin, A., Sackett-Taylor, A. C., & Forcier, M. (2020). Treatment paradigms for adolescents: Gender-affirming hormonal care. In A. Cohen-Kettenis, & F. J. G. Steensma (Eds.), *Pediatric gender identity: Gender-affirming care for transgender & gender diverse youth* (pp. 235–257). Springer Nature. 10.1007/978-3-030-38909-3_14

[CIT0029] Clark, B. A. (2021). Narratives of regret: Resisting cisnormative and bionormative biases in fertility and family creation counseling for transgender youth. *IJFAB: International Journal of Feminist Approaches to Bioethics*, *14*(2), 157–179. 10.3138/ijfab-14.2.09

[CIT0030] Clark, B. A., & Virani, A. (2021). “This wasn’t a split-second decision”: An empirical ethical analysis of transgender youth capacity, rights, and authority to consent to hormone therapy. *Journal of Bioethical Inquiry*, *18*(1), 151–164. 10.1007/s11673-020-10086-9 33502682 PMC8043901

[CIT0031] Cocchetti, C., Ristori, J., Romani, A., Maggi, M., & Fisher, A. D. (2020). Hormonal treatment strategies tailored to non-binary transgender individuals. *Journal of Clinical Medicine*, *9*(6), 1609. 10.3390/jcm9061609 32466485 PMC7356977

[CIT0032] Coleman, E., Radix, A. E., Bouman, W. P., Brown, G. R., de Vries, A. L. C., Deutsch, M. B., Ettner, R., Fraser, L., Goodman, M., Green, J., Hancock, A. B., Johnson, T. W., Karasic, D. H., Knudson, G. A., Leibowitz, S. F., Meyer-Bahlburg, H. F. L., Monstrey, S. J., Motmans, J., Nahata, L., … Arcelus, J. (2022). Standards of care for the health of transgender and gender diverse people, version 8. *International Journal of Transgender Health*, *23*(Suppl 1), S1–S259. 10.1080/26895269.2022.2100644 36238954 PMC9553112

[CIT0033] Cookson, R., & Dolan, P. (2000). Principles of justice in health care rationing. *Journal of Medical Ethics*, *26*(5), 323–329. 10.1136/jme.26.5.323 11055033 PMC1733291

[CIT0034] Costa, R., & Colizzi, M. (2016). The effect of cross-sex hormonal treatment on gender dysphoria individuals’ mental health: A systematic review. *Neuropsychiatric Disease and Treatment*, *12*, 1953–1966. 10.2147/NDT.S95310 27536118 PMC4977075

[CIT0035] Davis, S. A., & St. Amand, C. (2014). Effects of testosterone treatment and chest reconstruction surgery on mental health and sexuality in female-to-male transgender people. *International Journal of Sexual Health*, *26*(2), 113–128. 10.1080/19317611.2013.833152

[CIT0036] de Castro, C., Solerdelcoll, M., Plana, M. T., Halperin, I., Mora, M., Ribera, L., Castelo-Branco, C., Gómez-Gil, E., & Vidal, A. (2022). High persistence in Spanish transgender minors: 18 years of experience of the Gender Identity Unit of Catalonia. *Revista de Psiquiatría y Salud Mental*. Advance online publication. 10.1016/j.rpsm.2022.02.001 40603254

[CIT0037] de Lara, D. L., Rodríguez, O. P., Flores, I. C., Masa, J. L. P., Campos-Muñoz, L., Hernández, M. C., & Amador, J. T. R. (2020). Psychosocial assessment in transgender adolescents. *Anales de Pediatría (English Edition)*, *93*(1), 41–48. 10.1016/j.anpede.2020.01.004 32144041

[CIT0038] Dewey, J. M., & Gesbeck, M. M. (2017). (Dys) functional diagnosing: Mental health diagnosis, medicalization, and the making of transgender patients. *Humanity & Society*, *41*(1), 37–72. 10.1177/0160597615604651

[CIT0039] Durwood, L., Kuvalanka, K. A., Kahn-Samuelson, S., Jordan, A. E., Rubin, J. D., Schnelzer, P., Devor, A. H., & Olson, K. R. (2022). Retransitioning: The experiences of youth who socially transition genders more than once. *International Journal of Transgender Health*, *23*(4), 409–427. 10.1080/26895269.2022.2085224 36324883 PMC9621273

[CIT0040] Ehrensaft, D. (2011). *Gender born, gender made: Raising healthy gender-nonconforming children.* The Experiment.

[CIT0041] Ehrensaft, D. (2016). *The gender creative child: Pathways for nurturing and supporting children who live outside gender boxes.* The Experiment.

[CIT0042] Friesen, P., Kearns, L., Redman, B., & Caplan, A. L. (2017). Rethinking the Belmont report? *The American Journal of Bioethics: AJOB*, *17*(7), 15–21. 10.1080/15265161.2017.1329482 28661753

[CIT0043] Fuqua, J. S. (2013). Treatment and outcomes of precocious puberty: An update. *The Journal of Clinical Endocrinology and Metabolism*, *98*(6), 2198–2207. 10.1210/jc.2013-1024 23515450

[CIT0044] Gerritse, K., Hartman, L. A., Bremmer, M. A., Kreukels, B. P. C., & Molewijk, B. C. (2021). Decision-making approaches in transgender healthcare: Conceptual analysis and ethical implications. *Medicine, Health Care, and Philosophy*, *24*(4), 687–699. 10.1007/s11019-021-10023-6 34008081 PMC8557156

[CIT0045] Gill-Peterson, J. (2018). *Histories of the transgender child*. University of Minnesota Press.

[CIT0046] Giovanardi, G. (2017). Buying time or arresting development? The dilemma of administering hormone blockers in trans children and adolescents. *Porto Biomedical Journal*, *2*(5), 153–156. 10.1016/j.pbj.2017.06.001 32258611 PMC6806792

[CIT0047] Grannis, C., Leibowitz, S. F., Gahn, S., Nahata, L., Morningstar, M., Mattson, W. I., Chen, D., Strang, J. F., & Nelson, E. E. (2021). Testosterone treatment, internalizing symptoms, and body image dissatisfaction in transgender boys. *Psychoneuroendocrinology*, *132*(1), 105358. 10.1016/j.psyneuen.2021.105358 34333318

[CIT0048] Green, A. E., DeChants, J. P., Price, M. N., & Davis, C. K. (2022). Association of gender-affirming hormone therapy with depression, thoughts of suicide, and attempted suicide among transgender and nonbinary youth. *The Journal of Adolescent Health*, *70*(4), 643–649. 10.1016/j.jadohealth.2021.10.036 34920935

[CIT0049] Grimstad, F., & Boskey, E. (2020). How should decision-sharing roles be considered in adolescent gender surgeries? *AMA Journal of Ethics*, *22*(5), 452–457. 10.1001/amajethics.2020.452 32449665

[CIT0050] Hann, M., Ivester, R., & Denton, G. D. (2017). Bioethics in practice: Ethical issues in the care of transgender patients. *Ochsner Journal*, *17*(2), 144–145. 28638286 PMC5472072

[CIT0051] Hannema, S. E., Klink, D. T., den Heijer, M., & Wiepjes, C. M. (2022). Continuation of gender-affirming hormones in transgender people starting puberty suppression in adolescence: A cohort study in the Netherlands. *The Lancet. Child & Adolescent Health*, *6*(12), 869–875. 10.1016/S2352-4642(22)00254-1 36273487

[CIT0052] Hässler, T., Glazier, J. J., & Olson, K. R. (2022). Consistency of gender identity and preferences across time: An exploration among cisgender and transgender children. *Developmental Psychology*, *58*(11), 2184–2196. 10.1037/dev0001419 35951394 PMC9631298

[CIT0053] Healy, R. W., & Allen, L. R. (2019). Family therapy with transgender minors: A case study. *Clinical Social Work Journal*, *48*(4), 402–411. 10.1007/s10615-019-00704-4

[CIT0054] Hembree, W. C., Cohen-Kettenis, P. T., Gooren, L., Hannema, S. E., Meyer, W. J., Murad, M. H., Rosenthal, S. M., Safer, J. D., Tangpricha, V., & T’Sjoen, G. G. (2017). Endocrine treatment of gender-dysphoric/gender-incongruent persons: An endocrine society clinical practice guideline. *The Journal of Clinical Endocrinology and Metabolism*, *102*(11), 3869–3903. 10.1210/jc.2017-01658 28945902

[CIT0055] Holm, S. (1995). Not just autonomy—The principles of American biomedical ethics. *Journal of Medical Ethics*, *21*(6), 332–338. 10.1136/jme.21.6.332 8778456 PMC1376829

[CIT0056] Howard, D. (2022). Transformative choices and the specter of regret. *Journal of the American Philosophical Association*, *8*(1), 72–91. 10.1017/apa.2020.51

[CIT0057] Human Rights Campaign, American Academy of Pediatrics, & American College of Osteopathic Pediatricians. (2016). Supporting & caring for transgender children. https://www.hrc.org/resources/supporting-caring-for-transgender-children

[CIT0058] Huxtable, R. (2013). For and against the four principles of biomedical ethics. *Clinical Ethics*, *8*(2-3), 39–43. 10.1177/1477750913486245

[CIT0059] Ives, G. C., Fein, L. A., Finch, L., Sluiter, E. C., Lane, M., Kuzon, W. M., & Salgado, C. J. (2019). Evaluation of BMI as a risk factor for complications following gender-affirming penile inversion vaginoplasty. *Plastic and Reconstructive Surgery. Global Open*, *7*(3), e2097. 10.1097/GOX.0000000000002097 31044103 PMC6467628

[CIT0060] Johnson, T. W., & Irwig, M. S. (2014). The hidden world of self-castration and testicular self-injury. *Nature Reviews. Urology*, *11*(5), 297–300. 10.1038/nrurol.2014.84 24709968

[CIT0061] Kaltiala, R., Heino, E., Työläjärvi, M., & Suomalainen, L. (2020). Adolescent development and psychosocial functioning after starting cross-sex hormones for gender dysphoria. *Nordic Journal of Psychiatry*, *74*(3), 213–219. 10.1080/08039488.2019.1691260 31762394

[CIT0062] Kaltiala-Heino, R., Bergman, H., Työläjärvi, M., & Frisén, L. (2018). Gender dysphoria in adolescence: Current perspectives. *Adolescent Health, Medicine and Therapeutics*, *9*(9), 31–41. 10.2147/ahmt.s135432 29535563 PMC5841333

[CIT0063] Katz, A. L., Webb, S. A., Macauley, R. C., Mercurio, M. R., Moon, M. R., Okun, A. L., Opel, D. J., & Statter, M. B. (2016). Informed consent in decision-making in pediatric practice. *Pediatrics*, *138*(2), e20161485. 10.1542/peds.2016-1485 27456510

[CIT0064] Karrington, B. (2022). Defining desistance: Exploring desistance in transgender and gender expansive youth through systematic literature review. *Transgender Health*, *7*(3), 189–212. 10.1089/trgh.2020.0129 36643060 PMC9829142

[CIT0065] Kidd, K. M., Sequeira, G. M., Paglisotti, T., Katz-Wise, S. L., Kazmerski, T. M., Hillier, A., Miller, E., & Dowshen, N. (2021). “This could mean death for my child”: Parent perspectives on laws banning gender-affirming care for transgender adolescents. *The Journal of Adolescent Health*, *68*(6), 1082–1088. 10.1016/j.jadohealth.2020.09.010 33067153 PMC8041924

[CIT0066] Kimberly, L., Folkers, K. M., Friesen, P., Sultan, D., Quinn, G. P., Bateman-House, A., Parent, B., Konnoth, C., Janssen, A., Shah, L. D., Bluebond-Langner, R., & Salas-Humara, C. (2018). Ethical issues in gender-affirming care for youth. *Pediatrics*, *142*(6), e20181537. 10.1542/peds.2018-1537 30401789

[CIT0067] Kimberly, L., Folkers, K. M., Karrington, B., Wernick, J., Busa, S., & Salas-Humara, C. (2021). Navigating evolving ethical questions in decision making for gender-affirming medical care for adolescents. *The Journal of Clinical Ethics*, *32*(4), 307–321. 10.1086/JCE2021324307 34928859

[CIT0068] Korte, A., Lehmkuhl, U., Goecker, D., Beier, K. M., Krude, H., & Grüters-Kieslich, A. (2008). Gender identity disorders in childhood and adolescence: Currently debated concepts and treatment strategies. *Deutsches Arzteblatt International*, *105*(48), 834–841. 10.3238/arztebl.2008.0834 19578420 PMC2697020

[CIT0069] Krishna, K. B., Fuqua, J. S., Rogol, A. D., Klein, K. O., Popovic, J., Houk, C. P., Charmandari, E., Lee, P. A., Freire, A. V., Ropelato, M. G., Yazid Jalaludin, M., Mbogo, J., Kanaka-Gantenbein, C., Luo, X., Eugster, E. A., Klein, K. O., Vogiatzi, M. G., Reifschneider, K., Bamba, V., … Medina Bravo, P. G. (2019). Use of gonadotropin-releasing hormone analogs in children: Update by an international consortium. *Hormone Research in Paediatrics*, *91*(6), 357–372. 10.1159/000501336 31319416

[CIT0070] Kuper, L. E., Lindley, L., & Lopez, X. (2019). Exploring the gender development histories of children and adolescents presenting for gender affirming medical care. *Clinical Practice in Pediatric Psychology*, *7*(3), 217–228. 10.1037/cpp0000290

[CIT0071] Kuper, L. E., Stewart, S., Preston, S., Lau, M., & Lopez, X. (2020). Body dissatisfaction and mental health outcomes of youth on gender-affirming hormone therapy. *Pediatrics*, *145*(4), e20193006. 10.1542/peds.2019-3006 32220906

[CIT0072] Lee, J. Y., Finlayson, C., Olson-Kennedy, J., Garofalo, R., Chan, Y. M., Glidden, D. V., & Rosenthal, S. M. (2020). Low bone mineral density in early pubertal transgender/gender diverse youth: Findings from the Trans Youth Care Study. *Journal of the Endocrine Society*, *4*(9), bvaa065. 10.1210/jendso/bvaa065 32832823 PMC7433770

[CIT0073] Lim, A., Young, R. L., & Brewer, N. (2022). Autistic adults may be erroneously perceived as deceptive and lacking credibility. *Journal of Autism and Developmental Disorders*, *52*(2), 490–507. 10.1007/s10803-021-04963-4 33730319 PMC8813809

[CIT0074] MacKinnon, K. R., Ashley, F., Kia, H., Lam, J. S. H., Krakowsky, Y., & Ross, L. E. (2021). Preventing transition “regret”: An institutional ethnography of gender-affirming medical care assessment practices in Canada. *Social Science & Medicine (1982)*, *291*, 114477. 10.1016/j.socscimed.2021.114477 34666278

[CIT0075] McPhail, D., Rountree-James, M., & Whetter, I. (2016). Addressing gaps in physician knowledge regarding transgender health and healthcare through medical education. *Canadian Medical Education Journal*, *7*(2), e70–e78. 10.36834/cmej.36785 28344694 PMC5344057

[CIT0076] McQueen, P. (2017). The role of regret in medical decision-making. *Ethical Theory and Moral Practice*, *20*(5), 1051–1065. 10.1007/s10677-017-9844-8

[CIT0077] Mepham, N., Bouman, W. P., Arcelus, J., Hayter, M., & Wylie, K. R. (2014). People with gender dysphoria who self-prescribe cross-sex hormones: Prevalence, sources, and side effects knowledge. *The Journal of Sexual Medicine*, *11*(12), 2995–3001. 10.1111/jsm.12691 25213018

[CIT0078] Michel, A., Ansseau, M., Legros, J. J., Pitchot, W., & Mormont, C. (2002). The transsexual: What about the future? *European Psychiatry: The Journal of the Association of European Psychiatrists*, *17*(6), 353–362. 10.1016/S0924-9338(02)00703-4 12457746

[CIT0079] Mijaljica, G. (2014). Medical ethics, bioethics and research ethics education perspectives in South East Europe in graduate medical education. *Science and Engineering Ethics*, *20*(1), 237–247. 10.1007/s11948-013-9432-9 23436144

[CIT0080] New South Wales Ministry of Health. (2020). Consent to medical and healthcare treatment manual. https://www.health.nsw.gov.au/policies/manuals/Publications/consent-manual.pdf

[CIT0081] Olson, K. R., Durwood, L., Horton, R., Gallagher, N. M., & Devor, A. (2022). Gender identity 5 years after social transition. *Pediatrics*, *150*(2), e2021056082. 10.1542/peds.2021-056082 35505568 PMC9936352

[CIT0082] Olson, K. R., Key, A. C., & Eaton, N. R. (2015). Gender cognition in transgender children. *Psychological Science*, *26*(4), 467–474. 10.1177/0956797614568156 25749700

[CIT0083] Owen, G. S., Freyenhagen, F., Richardson, G., & Hotopf, M. (2009). Mental capacity and decisional autonomy: An interdisciplinary challenge. *Inquiry*, *52*(1), 79–107. 10.1080/00201740802661502

[CIT0084] Pellegrino, E. D. (1994). Patient and physician autonomy: Conflicting rights and obligations in the physician-patient relationship. *Journal of Contemporary Health Law & Policy*, *10*(1), 47–68. 10134815

[CIT0085] Priest, M. (2019). Transgender children and the right to transition: Medical ethics when parents mean well but cause harm. *The American Journal of Bioethics: AJOB*, *19*(2), 45–59. 10.1080/15265161.2018.1557276 30784385

[CIT0086] QueerDoc. (2022, November 21). Adding to your bottom line: Phallus-preserving vulvovaginoplasty. https://queerdoc.com/phallus-preserving-vaginoplasty/

[CIT0087] Quinn, G. P., Sampson, A., & Campo-Engelstein, L. (2018). Familial discordance regarding fertility preservation for a transgender teen: An ethical case study. *The Journal of Clinical Ethics*, *29*(4), 261–265. 10.1086/JCE2018294261 30605435

[CIT0088] Rao, G., Powell-Wiley, T. M., Ancheta, I., Hairston, K., Kirley, K., Lear, S. A., North, K. E., Palaniappan, L., & Rosal, M. C. (2015). Identification of obesity and cardiovascular risk in ethnically and racially diverse populations. *Circulation*, *132*(5), 457–472. 10.1161/CIR.0000000000000223 26149446

[CIT0089] Romero, K., & Reingold, R. (2013). Advancing adolescent capacity to consent to transgender-related health care in Colombia and the USA. *Reproductive Health Matters*, *21*(41), 186–195. 10.1016/S0968-8080(13)41695-6 23684201

[CIT0090] Rosenthal, S. M. (2014). Approach to the patient: Transgender youth: Endocrine considerations. *The Journal of Clinical Endocrinology and Metabolism*, *99*(12), 4379–4389. 10.1210/jc.2014-1919 25140398

[CIT0091] Ross, M. B., Wesseling, S., Mullender, M., Kreukels, B. P., & van de Grift, T. (2023). Expectations and experienced outcomes regarding gender-affirming surgeries: A pilot study. *Transgender Health*. Advance online publication. 10.1089/trgh.2022.0118 PMC11456761 39385959

[CIT0092] Sansfaçon, A. P., Gelly, M. A., Gravel, R., Medico, D., Baril, A., Susset, F., & Paradis, A. (2023). A nuanced look into youth journeys of gender transition and detransition. *Infant and Child Development*, *32*(2), e2402. 10.1002/icd.2402

[CIT0093] Shea, M. (2020a). Forty years of the four principles: Enduring themes from Beauchamp and Childress. *The Journal of Medicine and Philosophy: A Forum for Bioethics and Philosophy of Medicine*, *45*(4-5), 387–395. 10.1093/jmp/jhaa020

[CIT0094] Shea, M. (2020b). Principlism’s balancing act: Why the principles of biomedical ethics need a theory of the good. *The Journal of Medicine and Philosophy*, *45*(4-5), 441–470. 10.1093/jmp/jhaa014 32726809

[CIT0095] Shumer, D. E., & Tishelman, A. C. (2015). The role of assent in the treatment of transgender adolescents. *The International Journal of Transgenderism*, *16*(2), 97–102. 10.1080/15532739.2015.1075929 27175107 PMC4861162

[CIT0096] Shuster, S. M. (2016). Uncertain expertise and the limitations of clinical guidelines in transgender healthcare. *Journal of Health and Social Behavior*, *57*(3), 319–332. 10.1177/0022146516660343 27601408

[CIT0097] Snow, A., Cerel, J., & Frey, L. (2022). A safe bet? Transgender and gender diverse experiences with inclusive therapists. *The American Journal of Orthopsychiatry*, *92*(2), 154–158. 10.1037/ort0000599 34941294

[CIT0098] St. Amand, C. M., & Ehrensaft, D. (2018). *The gender affirmative model: An interdisciplinary approach to supporting transgender and gender expansive children.* American Psychological Association.

[CIT0099] Stelmar, J., Smith, S. M., Lee, G., Zaliznyak, M., & Garcia, M. M. (2023). Shallow-depth vaginoplasty: Preoperative goals, postoperative satisfaction, and why shallow-depth vaginoplasty should be offered as a standard feminizing genital gender-affirming surgery option. *The Journal of Sexual Medicine*, *20*(11), 1333–1343. 10.1093/jsxmed/qdad111 37721184

[CIT0100] Strand, N. K., & Jones, N. L. (2021). Invisibility of “gender dysphoria.” *Medicine and Society*, *23*(7), e.557–e.562. 10.1001/amajethics.2021.557 34351266

[CIT0101] Taormina, J. M., Gilden, A. H., & Iwamoto, S. J. (2023). Meeting the body mass index requirement for gender-affirming surgery using antiobesity medication. *JCE Case Reports*, *1*(3), luad067. 10.1210/jcemcr/luad067 PMC10301860 37388627

[CIT0102] Temple Newhook, J., Pyne, J., Winters, K., Feder, S., Holmes, C., Tosh, J., Sinnott, M.-L., Jamieson, A., & Pickett, S. (2018). A critical commentary on follow-up studies and “desistance” theories about transgender and gender-nonconforming children. *International Journal of Transgenderism*, *19*(2), 212–224. 10.1080/15532739.2018.1456390

[CIT0103] Thompson, L. (2019). The conservative war on transgender rights has reached a new low. https://www.motherjones.com/politics/2019/12/the-conservative-war-on-transgender-rights-has-reached-a-new-low/

[CIT0104] Tordoff, D. M., Wanta, J. W., Collin, A., Stepney, C., Inwards-Breland, D. J., & Ahrens, K. (2022). Mental health outcomes in transgender and nonbinary youths receiving gender-affirming care. *JAMA Network Open*, *5*(2), e220978. 10.1001/jamanetworkopen.2022.0978 35212746 PMC8881768

[CIT0105] Turban, J. L., King, D., Kobe, J., Reisner, S. L., & Keuroghlian, A. S. (2022). Access to gender-affirming hormones during adolescence and mental health outcomes among transgender adults. *PLoS One*, *17*(1), e0261039. 10.1371/journal.pone.0261039 35020719 PMC8754307

[CIT0106] Turban, J. L., Loo, S. S., Almazan, A. N., & Keuroghlian, A. S. (2021). Factors leading to “detransition” among transgender and gender diverse people in the United States: A mixed-methods analysis. *LGBT Health*, *8*(4), 273–280. 10.1089/lgbt.2020.0437 33794108 PMC8213007

[CIT0107] United Nations. (1989). Convention on the rights of the child. https://www.ohchr.org/en/professionalinterest/pages/crc.aspx

[CIT0108] United Nations Educational, Scientific, and Cultural Organization. (2016). Bioethics core curriculum. Section 1: Syllabus. https://unesdoc.unesco.org/ark:/48223/pf0000246885

[CIT0109] Varkey, B. (2021). Principles of clinical ethics and their application to practice. *Medical Principles and Practice*, *30*(1), 17–28. 10.1159/000509119 32498071 PMC7923912

[CIT0110] Vincent, B. (2018). Studying trans: Recommendations for ethical recruitment and collaboration with transgender participants in academic research. *Psychology & Sexuality*, *9*(2), 102–116. 10.1080/19419899.2018.1434558

[CIT0111] Vincent, B. (2019). Breaking down barriers and binaries in trans healthcare: The validation of non-binary people. *The International Journal of Transgenderism*, *20*(2-3), 132–137. 10.1080/15532739.2018.1534075 32999601 PMC6831034

[CIT0112] Vrouenraets, L. J., de Vries, A. L., de Vries, M. C., van der Miesen, A. I., & Hein, I. M. (2021). Assessing medical decision-making competence in transgender youth. *Pediatrics*, *148*(6), e2020049643. 10.1542/peds.2020-049643 34850191

[CIT0113] Walsh, R. J., Krabbendam, L., Dewinter, J., & Begeer, S. (2018). Brief report: Gender identity differences in autistic adults: Associations with perceptual and socio-cognitive profiles. *Journal of Autism and Developmental Disorders*, *48*(12), 4070–4078. 10.1007/s10803-018-3702-y 30062396

[CIT0114] Watson, L. B., Allen, L. R., Flores, M. J., Serpe, C., & Farrell, M. (2019). The development and psychometric evaluation of the Trans Discrimination Scale: TDS-21. *Journal of Counseling Psychology*, *66*(1), 14–29. 10.1037/cou0000301 30035589

[CIT0115] Wattel, L. L., Walsh, R. J., & Krabbendam, L. (2022). Theories on the link between autism spectrum conditions and trans gender modality: A systematic review. *Review Journal of Autism and Developmental Disorders*, *48*, 4070–4078. 10.1007/s40489-022-00338-2 PMC11127869 38803560

[CIT0116] Weithorn, L. A., & Campbell, S. B. (1982). The competency of children and adolescents to make informed treatment decisions. *Child Development*, *53*(6), 1589–1598. 10.2307/1130087 7172783

[CIT0117] Wenner, D. M., & George, B. R. (2021). Not just a tragic compromise: The positive case for adolescent access to puberty-blocking treatment. *Bioethics*, *35*(9), 925–931. 10.1111/bioe.12929 34427939

[CIT0118] Woodhouse, B. B. (2003). Re-visioning rights for children. In P. B. Pufall & R. P. Unsworth (Eds.), *Rethinking childhood* (pp. 229–243). Rutgers University Press. 10.36019/9780813558325-016

[CIT0119] World Health Organization. (2009). Research ethics committees: Basic concepts for capacity-building. http://www.who.int/ethics/Ethics_basic_concepts_ENG.pdf

[CIT0120] World Health Organization. (2015). Global health ethics key issues: Global network of WHO collaborating centres for bioethics. https://apps.who.int/iris/handle/10665/164576

[CIT0121] Xu, J. Y., O’Connell, M. A., Notini, L., Cheung, A. S., Zwickl, S., & Pang, K. C. (2021). Selective estrogen receptor modulators: A potential option for non-binary gender-affirming hormonal care? *Frontiers in Endocrinology*, *12*, 701364. 10.3389/fendo.2021.701364 34226826 PMC8253879

[CIT0122] Zaliznyak, M., Bresee, C., & Garcia, M. M. (2020). Age at first experience of gender dysphoria among transgender adults seeking gender-affirming surgery. *JAMA Network Open*, *3*(3), e201236. 10.1001/jamanetworkopen.2020.1236 32176303 PMC7076333

